# Calcium-Sensing Receptor Gene: Regulation of Expression

**DOI:** 10.3389/fphys.2016.00394

**Published:** 2016-09-13

**Authors:** Geoffrey N. Hendy, Lucie Canaff

**Affiliations:** Experimental Therapeutics and Metabolism, McGill University Health Centre-Research Institute, Departments of Medicine, Physiology, and Human Genetics, McGill UniversityMontréal, QC, Canada

**Keywords:** gene, alternative transcripts, transcription, vitamin D, proinflammatory cytokines, glial cells missing-2, DNA methylation, microRNA

## Abstract

The human calcium-sensing receptor gene (*CASR*) has 8 exons, and localizes to chromosome 3q. Exons 1A and 1B encode alternative 5′-untranslated regions (UTRs) that splice to exon 2 encoding the AUG initiation codon. Exons 2–7 encode the CaSR protein of 1078 amino acids. Promoter P1 has TATA and CCAAT boxes upstream of exon 1A, and promoter P2 has Sp1/3 motifs at the start site of exon 1B. Exon 1A transcripts from the P1 promoter are reduced in parathyroid tumors and colon carcinomas. Studies of colon carcinomas and neuroblastomas have emphasized the importance of epigenetic changes—promoter methylation of the GC-rich P2 promoter, histone acetylation—as well as involvement of microRNAs in bringing about *CASR* gene silencing and reduced CaSR expression. Functional cis-elements in the *CASR* promoters responsive to 1,25-dihydroxyvitamin D [1,25(OH)_2_D], proinflammatory cytokines, and the transcription factor glial cells missing-2 (GCM2) have been characterized. Reduced levels of CaSR and reduced responsiveness to active vitamin D in parathyroid neoplasia and colon carcinoma may blunt the “tumor suppressor” activity of the CaSR. The hypocalcemia of critically ill patients with burn injury or sepsis is associated with *CASR* gene upregulation by TNF-alpha and IL-1beta via kappaB elements, and by IL-6 via Stat1/3 and Sp1/3 elements in the *CASR* gene promoters, respectively. The *CASR* is transactivated by GCM2—the expression of which is essential for parathyroid gland development. Hyperactive forms of GCM2 may contribute to later parathyroid hyperactivity or tumorigenesis. The expression of the CaSR—the calciostat—is regulated physiologically and pathophysiologically at the gene level.

## The *CASR* gene

The single-copy *CASR* gene that maps to human 3q13.3-21 encodes a Class C G protein-coupled receptor family member (Pollak et al., 1993; Janicic et al., 1995). The T-cell antigen *CD86* gene lies upstream and the cysteine protease inhibitor *CSTA* gene downstream of the *CASR* gene and all are transcribed in the same 5′ to 3′ direction.

**Figure 1:**
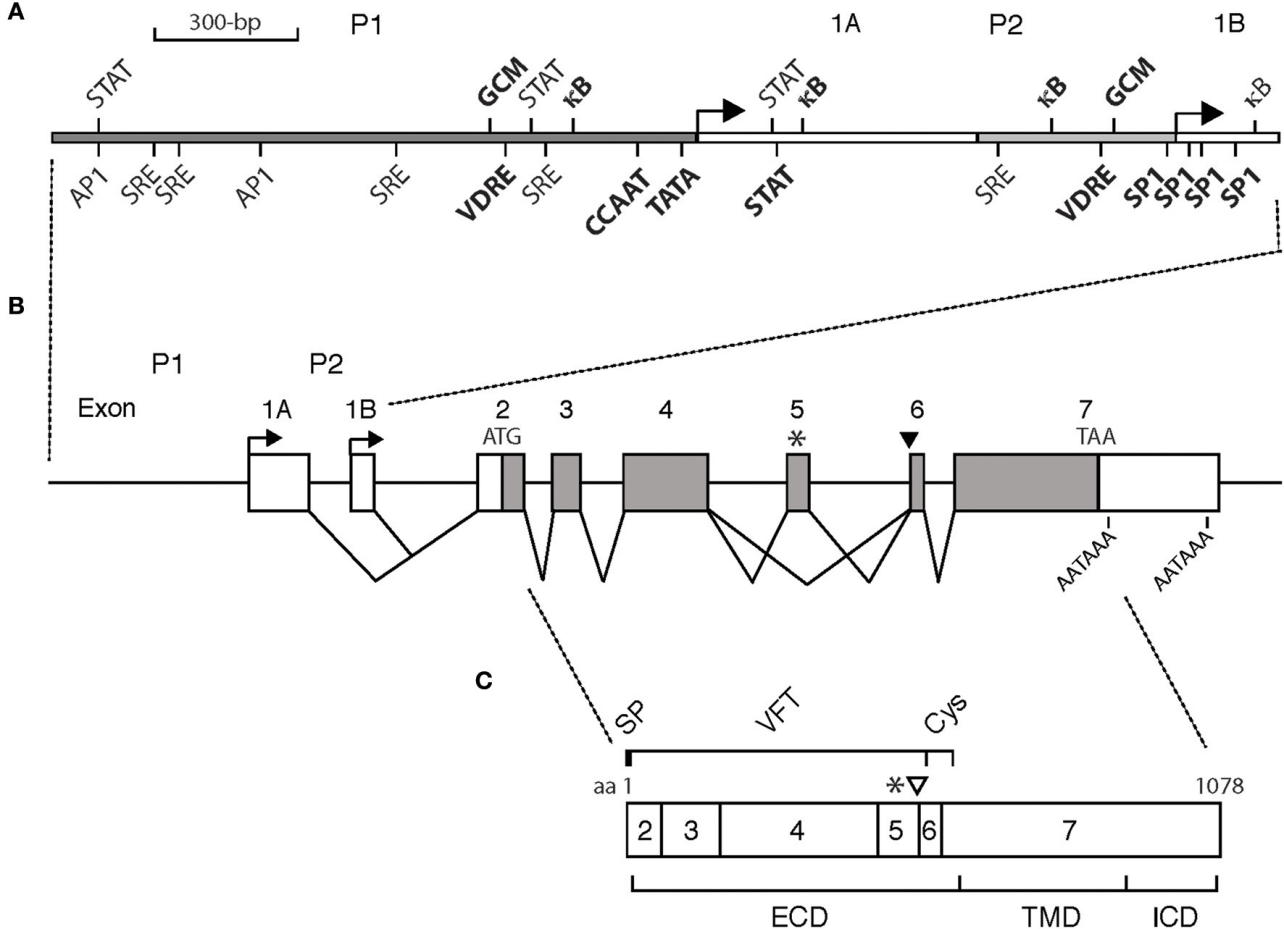
**Schematic of (A) the *CASR* gene promoters, (B) the *CASR* gene exon/intron organization, and (C) CaSR protein. (A)** Promoter P1 and P2, gray bars. Exons 1A and 1B, white bars. Transcription start sites, arrows. CCAAT and TATA boxes, and SP-1 sites driving transcription of exon 1A and 1B, respectively, are bolded. Cis-acting elements are shown. VDRE, vitamin D response element; κB, kappa-B element responsive to nuclear factor kappa-light-chain-enhancer of activated B cells; STAT, signal transducer and activator of transcription; GCM, glial cells missing; AP1, activator protein 1; SRE, serum response element. Bolded: those shown to be functionally active. Not bolded: those predicted but either not functionally active or not yet evaluated. Not all predicted cis-acting elements are shown. **(B)** Exon/intron organization of the *CASR* gene. Exons are drawn to scale introns are not. White bars: mRNA untranslated (exons; 1A, 1B, part of 2, part of 7). Gray bars: mRNA protein coding (exons; part of 2, 3-6, part of exon 7). ATG: initiation codon. TAA: stop codon. AATAAA: polyadenylation signals. Alternative splicing of exons 1A and 1B to exon 2 is shown. Asterisk, ^*^: alternative transcript lacking exon 5. Black arrowhead: alternative transcript having additional 30 bases at the beginning of exon 6. **(C)** CaSR protein: 1078 amino acid (aa) protein encoded by exons 2-7. Asterisk, ^*^: minus 77 aa encoded by exon 5. Open arrowhead: additional 10 aa encoded by extra 30 bases of alternative RNA transcript. SP, signal peptide; VFT, venus flytrap domain; Cys, cysteine rich domain; ECD, extracellular domain; TMD, transmembrane domain; ICD, intracellular domain.

The *CASR* gene has eight exons and spans ~100-kb (Yun et al., 2007) (Figure 1). Exons 2 to 7 encode the CaSR protein of 1078 aa (GenBank #U20759). Two different polyadenylation signal sequences within exon 7 may be used, to generate either a short (177-nucleotide) or a long (1304-nucleotide) 3′-untranslated region (UTR) (Aida et al., 1995; Garrett et al., 1995). Exon 2 encodes 242 nucleotides of the 5′-UTR upstream of the ATG translation initiation codon. Exons 1A and 1B encode alternative 5′-UTRs that splice to a common segment encoded by exon 2 (Garrett et al., 1995; Chikatsu et al., 2000).

This organization of exons, conserved around primary protein domains, is first seen in aquatic vertebrates (Naito et al., 1998). With diversification from the teleost fishes to tetrapods, mammals, and primates, the evolutionary changes have been greatest in the 5′ and 3′ domains. While overall exonic structure is preserved a striking increase in intron size has occurred from teleost fish to higher species (Loretz, 2008). Linkage disequilibrium analysis across the human gene shows a central haploblock extending from exon 2 to 7 that is distinct from separate haploblocks for genetic variants in the 5′ and 3′ flanking regions (Yun et al., 2007).

Thus, the mouse, rat and human genes are organized in a similar manner. Both rodent genes comprise at least 7 exons and the translational start site is in exon 2. Alignment of expressed sequence tags (ESTs) and cloned rodent cDNAs reveal that, just like the human *CASR* gene, mouse and rat *CASR* genes have at least 2 distinct 5′UTRs (Exons 1A and 1B), suggesting the presence of at least 2 promoters. The mouse and rat *CASR* genes share >85% nucleotide identity (exons and introns) and 40% with the human *CASR*. The VDREs and κB elements (see below) that we have characterized in our studies of the human *CASR* gene promoters are conserved in the rodent *Casr* promoters.

### Alternative transcripts

The *CASR* gene is highly expressed in the parathyroid gland and renal tubule (Brown et al., 1993). However, the gene is widely expressed at lower levels in other tissues, for example in liver (Canaff et al., 2001), bone (Goltzman and Hendy, 2015) and in lung, breast, placenta, vasculature and gut (Brennan et al., 2013). Several alternative transcripts have been identified, raising the question of their function and regulation of their expression.

Coexistence of transcripts encoding the same 1078 amino acid protein in human parathyroid but different 5′ UTRs suggested alternative splicing (Garrett et al., 1995). Moreover, the transcripts may have either a short or long 3′ UTR. One low abundance transcript encodes an additional 10 amino acid stretch inserted after amino acid 536 in the exodomain (GenBank #U20760) but does not alter CaSR function (Garrett et al., 1995). The predominant transcript is 5.4-kb in length, while less abundant transcripts of 10, 4.8, and 4.2-kb are also found (Garrett et al., 1995). In human kidney, the 5.4-kb transcript is similarly predominant with the less abundant 10-kb transcript also present (Aida et al., 1995).

The human *CASR* gene has two promoters driving transcription of alternative 5′ UTR exons (1A and 1B) (Chikatsu et al., 2000). By northern blot analysis of human parathyroid adenomas and normal glands, the 5.4 and 10-kb transcripts (see above) appeared to be exclusive to exon 1A use, while the 4.2-kb transcripts are derived from either 1A or 1B (Chikatsu et al., 2000). Real-time PCR (qPCR) analysis of human parathyroid cells revealed that exon 1B-transcripts were much more highly expressed than exon 1A-containing transcripts (Mizobuchi et al., 2009). Transcripts of the 5.4 and 4.2-kb size derive from use of the two alternative polyadenylation sites in the 3′ UTR tract (Chikatsu et al., 2000).

An exon 3-deleted CASR transcript has been reported in thyroid TT cells (Freichel et al., 1996), in placental cytotrophoblast (Bradbury et al., 1998), and in parathyroid, thyroid, and kidney (D'souza-Li et al., 2001). Fusion of exon 2 to exon 4 results in a truncated protein that is poorly expressed and not trafficked to the cell surface.

In human keratinocytes, an alternatively spliced transcript lacking exon 5 encodes a variant CaSR with a 77-amino acid in-frame deletion in the exodomain (Oda et al., 1998). This variant exerts a dominant negative effect on the full-length protein making it less responsive to calcium. In addition, the relative amounts of full-length vs. alternatively spliced transcript decrease during keratinocyte differentiation (Oda et al., 1998).

Consideration of alternatively spliced forms are of importance in phenotype evaluation of *Casr* mice knocked out by deletion of exon 5 (Ho et al., 1995). In the growth plate (Rodriguez et al., 2005), skin (Oda et al., 2000) and kidney (Oda et al., 2000) of the knockout mice, the *Casr* message lacking exon 5 is upregulated and compensates for the absence of the full-length counterpart in bone and cartilage (Rodriguez et al., 2005). Studies of *Casr* knockouts in which both full-length and exon 5 transcripts are deleted have suggested that the exon 5-deletion model might be hypomorphic with respect to CaSR actions in the skeleton (Chang et al., 2008).

### Transcriptional control of the *CASR* gene

Regulated *CASR* gene expression is important in growth and development (Riccardi et al., 2013), and in normal adult physiology (Brown, 2013) and in disease pathogenesis (Hannan and Thakker, 2013). Some of the factors and mechanisms involved in transactivation of the *CASR* gene have been identified (Hendy et al., 2013).

Human *CASR* transcription is driven by either promoter P1, with a TATA box at nucleotide −26 and a CCAAT box at −110 relative to the start site, or P2 with an Sp1/3 site at the transcriptional start site (Figure 1). The rat and mouse *Casr* genes have a similar organization. Both promoters drive significant levels of basal activity, with promoter P2 being 2.5-fold more active than P1 in most cell types examined as assessed by transfected promoter-reporter analysis (Canaff and Hendy, 2002). Also nuclear run-on assays that directly measure transcripts suggest greater exon 1B relative to exon 1A transcripts in human thyroid C-cells and renal proximal tubular cells. The presence of multiple promoters provides the potential for tissue-specific and/or developmental/temporal-specific regulated expression from one promoter vs. responsiveness to hormonal or nutritional stimuli from the other. Evidence for this has yet to be fully realized for the *CASR* gene. In fact, from studies done so far the opposite seems to be the case in that both promoters respond to active vitamin D, cytokines, and the parathyroid cell-specific regulator, GCM2 (see below). However, pathophysiological differences in usage of the *CASR* gene promoters have been suggested.

Chikatsu et al. (2000) showed the expression of exon 1A transcripts driven by the upstream promoter (P1), was decreased in adenomas, whereas expression of exon 1B transcripts driven by the (normally stronger) promoter P2 was unchanged. These results suggested that P1 activity was reduced in parathyroid adenomas and is qualitatively in agreement with several studies showing a decrease in CaSR mRNA and protein expression in parathyroid adenomas relative to normal glands (Kifor et al., 1996; Farnebo et al., 1997; Gogusev et al., 1997; Cetani et al., 2000; Corbetta et al., 2000). However, other mechanisms yet to be identified are likely to contribute to the greater relative decreases in mRNA and protein expression that are not accounted for quantitatively by the ~50% decrease of the apparently weaker P1 promoter. In a study of colorectal tumors, Kállay et al. (2003) found exon 1A transcript expression to be greater in the adjacent “normal” mucosa of colon cancer patients than in the tumor itself, and a highly significant decrease of exon 1A expression was found during progression from well-differentiated to poorly differentiated cancers whereas exon 1B transcript expression was not significantly altered. More recent studies have found hypermethylation of promoter P2 that drives the exon 1B transcripts (Fetahu et al., 2014a) and therefore reduced exon 1B transcript levels would be predicted. Further studies will be needed to resolve this apparent discrepancy.

### Vitamin D

Active vitamin D increases CaSR expression and this has been documented in several studies in rodents. Parathyroid CaSR mRNA was reduced by 40% in vitamin D-depleted relative to replete rats, and 1,25(OH)_2_D_3_ administration to vitamin D-replete rats enhanced parathyroid and kidney CaSR mRNA levels further (Brown et al., 1996). In another study administration of 1,25(OH)_2_D_3_ to rats upregulated renal CaSR mRNA levels in a dose-and time-dependent manner (Yao et al., 2005). Kidney CaSR expression (microarray analysis) was downregulated in VDR null mice relative to their wildtype littermates (Li et al., 2003) and likewise (ribonuclease protection assay) for *Cyp27*−/− mice lacking the 25-hydroxyvitamin D-1α-hydroxylase enzyme (Canaff et al., 2009a). Injection of 1,25(OH)_2_D_3_ in either normal mice or the *Cyp27b1*−/− mice caused upregulation of CaSR expression in parathyroid/thyroid and kidney (Li et al., 2003; Canaff et al., 2009a). Nuclear run-on assays that measure RNA transcripts showed that upregulation in thyroid and kidney cells occurred via gene transcription (Canaff and Hendy, 2002). Functional vitamin D response elements (VDREs) are present in both promoters, P1 and P2, of the human *CASR* gene (Canaff and Hendy, 2002) and are conserved in the rodent (Hendy et al., 2013). The VDREs of the *CASR* are typical in that they consist of two 6-bp half-sites separated by 3-bp that are bound by the vitamin D receptor (VDR)-retinoic acid X receptor (RXR) dimer. However, the VDREs are atypical in that the orientation of the half-sites is inverted to that which is normally found. VDREs of this type are found in the 25-hydroxyvitamin D-24-hydroxylase (*CYP24A1*) gene.

In the parathyroid gland, the 1,25(OH)_2_D-upregulated CaSR makes the gland more responsive to extracellular Ca^2+^ and PTH secretion is reduced. The negative action of extracellular Ca^2+^ on PTH synthesis and secretion and parathyroid cell proliferation is reinforced by the active vitamin D metabolite. Impaired extracellular Ca^2+^-sensing that drives increased parathyroid cell proliferation may contribute to parathyroid neoplasia. Somatic *CASR* mutations are rare, but parathyroid glands of patients with primary or severe uremic secondary hyperparathyroidism often have reduced CaSR expression (Kifor et al., 1996; Cetani et al., 2000; Corbetta et al., 2000). Thus, reduction in components of the vitamin D system, active vitamin D ligand either circulating levels or produced by parathyroid intracrine action, and/or target VDR levels, that normally decrease PTH synthesis and secretion, could play an additional role by negating the normal inhibitory effects of extracellular Ca^2+^ on PTH via decreased CaSR expression.

The activated kidney CaSR that can act independently of PTH to directly determine the circulating Ca^2+^ concentration (Loupy et al., 2012) inhibits the paracellular uptake of cations in the cortical thick ascending limb of the distal nephron and promotes hypercalciuria. Autosomal dominant hypocalcemia type 1 (ADH1) patients are heterozygous for a gain-of-function mutant *CASR*. Treatment of ADH1 patients with active vitamin D upregulates the oversensitive renal CaSR stimulating Ca^2+^ excretion provoking nephrocalcinosis, nephrolithiasis, and potential renal damage (Pearce et al., 1996; Lienhardt et al., 2001). For such patients a better treatment option is vitamin D itself whereby the amount 1,25(OH)_2_D formed is limited by product inhibition of the 25-hydroxyvitamin D-1alpha-hydroxylase enzyme in the proximal tubule (Obermannova et al., 2016). In some genetic forms of hypercalciuria, altered regulation of CaSR expression by vitamin D metabolites may be a critical factor contributing to stone formation. In a genetic stone-forming rat model of hypercalciuric nephrolithiasis, nephron VDR levels are elevated, CaSR levels are increased and calcium reabsorption reduced (Yao et al., 2005; Bai and Favus, 2006).

Alterations of CaSR expression have been implicated not only in hyperparathyroidism but also other neoplasms. The CaSR is expressed in human colon epithelium and regulates cell proliferation and differentiation. Cells of the colon crypt acquire CaSR expression as they differentiate and migrate toward the apex of the crypt (Chakrabarty et al., 2005). CaSR expression is weak or absent in colon carcinomas and is inversely correlated with differentiation status. Extracellular Ca^2+^ and 1,25(OH)_2_D upregulate *CASR* transcription and cyclin-dependent kinase inhibitor expression in the colon and Ca^2+^ and 1,25(OH)_2_D may exert their chemopreventative actions with respect to colon cancer, in part, through the CaSR (Chakrabarty et al., 2005). Fetahu et al. (2014b) demonstrated the upregulation of CaSR expression by 1,25(OH)_2_D_3_ in colon cancer cells. This would be consistent with the active vitamin D metabolite exerting its antiproliferative, prodifferentiating effects in part by inducing expression of the tumor suppressor, CaSR.

Vascular calcification occurs during aging and pathologically in atherosclerosis and chronic kidney disease. There is an inverse relationship between vascular calcification and expression of the CaSR (Alam et al., 2009) and maintenance of its expression can protect against calcification (Molostvov et al., 2015). Treatment of vascular smooth muscle cells with active vitamin D increases CaSR mRNA and protein levels (Mary et al., 2015). This would be consistent with 1,25(OH)_2_D transactivation of the *CASR* gene via its VDREs (Canaff and Hendy, 2002). Treatment with cinacalcet that enhances sensitivity of the CaSR to Ca^2+^ promotes CaSR expression at the cell surface while overall CaSR expression is not altered (Hénaut et al., 2014). This would be consistent with ligand induced trafficking of the CaSR to the cell surface (Breitwieser, 2013) and Ca^2+^ within the normal range and higher having no marked effect on *CASR* gene expression (see below for further detail of potential regulation of *CASR* by extracellular Ca^2+^).

### Calcium

In addition to 1,25(OH)_2_D, a potential regulator of *CASR* gene expression is extracellular Ca^2+^ itself. Initial studies did not find an effect of extracellular Ca^2+^ on parathyroid gland or whole kidney CaSR mRNA in the rat *in vivo* (Rogers et al., 1995; Brown et al., 1996). A lack of effect on *CASR* expression by circulating Ca^2+^ is perhaps not unexpected in tissues such as parathyroid gland or kidney, where the CaSR plays a critical role in Ca^2+^ homeostasis as a calciostat to sense small changes in extracellular Ca^2+^ concentration. Even slight alterations in the extracellular Ca^2+^ set-point (the extracellular Ca^2+^ concentration at which PTH secretion from the parathyroid gland or calcium reabsorption across the kidney tubule is half-maximal) brought about by changes in CaSR synthesis could disturb overall calcium homeostasis.

CaSR expression did not change in rats having altered circulating calcium levels as a result of infusion of CaCl_2_ or fed vitamin D-deficient diets supplemented with Ca^2+^ (Rogers et al., 1995; Brown et al., 1996). In these two studies the extracellular calcium levels ranged from 0.7 to 1.9 mM. However, potentially, lower or higher Ca^+^ concentrations could regulate CaSR expression and perhaps in a tissue-specific manner.

*CASR* gene transcription was modulated by extracellular Ca^+^ in studies of transfected *CASR* promoter-reporter constructs in human kidney proximal tubule cells and mouse distal convoluted tubule cells (Canaff, 2004). In both cell types, when the concentration of Ca^2+^ in the media was varied from 1 to 5 mM, there was only a slight increase in P1 and P2 promoter activity. However, there was markedly reduced transcriptional activity of P1 (by 50%) and P2 (by 30%) in both proximal and distal convoluted tubule cell types when cultured in a very low (0.25 mM) Ca^2+^ concentration. Therefore, from normal to high Ca^2+^ there was little regulation but from normal to low calcium there was significant regulation. The results from these *in vitro* experiments would be consistent with the lack of obvious effect of circulating Ca^2+^ in the *in vivo* studies referred to above (Rogers et al., 1995; Brown et al., 1996). In other cells—differentiated human colon carcinoma cells—upregulation of the CaSR occurred when the Ca^2+^ concentration of the culture medium was increased from very low to normal (Chakrabarty et al., 2005). Stimulatory effects of Ca^2+^ and 1,25(OH)_2_D were additive for promoter P2 but not for promoter P1 (Chakrabarty et al., 2005).

What would be the significance in the kidney of a reduced CaSR expression at low extracellular Ca^2+^ concentration? A decrease of CaSR expression in the proximal tubule would decrease the effect of calcium on the 25-hydroxyvitamin D-1alpha-hydroxylase activity i.e., the negative impact of Ca^2+^ in decreasing enzyme activity would be minimized. In the ascending limb and distal convoluted tubule, the combination of a reduced CaSR expression and a low extracellular Ca^2+^ would favor maximal Ca^2+^ reabsorption from the tubule lumen and allow PTH to stimulate Ca^2+^ reabsorption without being antagonized by the CaSR.

The precise pathways by which extracellular Ca^2+^ might transactivate the *CASR* gene are not known. If the extracellular Ca^2+^ effect is mediated via the CaSR then a transcription factor activated by mitogen activated protein kinase (MAPK) (e.g., Elk1) could be involved. In addition, the involvement of transcription factors in the reduced expression of the CaSR in neoplasia of parathyroid and colon, for example, are not known. In an *in vivo* murine model of primary hyperparathyroidism in which cyclin D1 overexpression is targeted to the parathyroid, expression of the CaSR in the gland is markedly reduced and correlates with the severity of the hyperparathyroidism (Kawata et al., 2005). Treatment of the mice with the allosteric CaSR activator cinacalcet to mimic high levels of extracellular Ca^2+^ suppressed serum PTH and Ca^2+^ and parathyroid cell proliferation but had no effect on the levels of parathyroid CaSR mRNA (Imanishi et al., 2011). This reinforces the view that normal or higher extracellular Ca^2+^ concentrations do not significantly modulate parathyroid CaSR expression levels.

### Proinflammatory cytokines

Evidence has accumulated that the increased levels of circulating cytokines such as IL-1β and IL-6 occurring in conditions of inflammation could provoke altered systemic Ca^2+^ homeostasis by modulating the setting of the calciostat—the expression of the CaSR. In critically ill patients such as those with sepsis and major burn injury hypocalcemia is common (Zaloga, 1992; Zivin et al., 2001; Steele et al., 2013) and in non-acutely ill patients undergoing surgery (Lepage et al., 1999). Serum IL-1β and IL-6 levels increase within hours of severe burns and infection (Kowal-Vern et al., 1994; Klein et al., 1995; Caldwell et al., 1997), and are inversely related to the falls in serum Ca^2+^ concentration (Lind et al., 2000) that may correlate with a poor prognosis (Guo et al., 1990; Nijsten et al., 1991; Schlüter et al., 1991; Ohzato et al., 1993; Yamada et al., 1996; Remick et al., 2002). Several factors such as decreased secretion of PTH and/or resistance to PTH action in kidney and bone may contribute to the hypocalcemia (Katz et al., 1992; Klein et al., 1997). In addition, the metabolism and action of vitamin D can be impaired. Calcitonin precursors are increased in the circulation of critically ill patients with sepsis (Lind et al., 2000; Müller et al., 2000; Becker et al., 2004, 2010). Certainly altered PTH secretion and action and vitamin D metabolism will be most important, whereas calcitonin plays a minor role in Ca^2+^ homeostatic control in human.

In patients with rheumatoid arthritis impaired secretion of PTH is inversely related to the inflammatory activity (Ekenstam et al., 1990). PTH secretion from bovine parathyroid cells was suppressed by IL-6 *in vitro* (Carlstedt et al., 1999) and the same effect was demonstrated for clinically relevant doses of IL-1β acting specifically via the IL-1 receptor in bovine parathyroid tissue slices *in vitro* with the added observation of upregulation of CaSR mRNA levels (Nielsen et al., 1997). Parathyroid CaSR mRNA levels were upregulated in an *in vivo* sheep model of burn injury in which increased circulating cytokine levels would occur (Murphey et al., 2000). Some proinflammatory cytokines can stimulate (rather than inhibit) the release of PTH. The IL-8B receptor (CXCR2) was identified in the bovine parathyroid gland (Angeletti et al., 1998). Cultured bovine parathyroid cells responded to IL-8 with increases in PTH mRNA levels and PTH secretion, although any role of altered CaSR expression in these effects is as yet unknown.

Dysregulated PTH release and altered circulating Ca^2+^ levels occurs in septic horses (Toribio et al., 2001; Hurcombe et al., 2009). Equine parathyroid cells in culture responded to elevations in extracellular Ca^2+^ (0.8–2 mM) with decreased PTH mRNA while CaSR mRNA expression was unaltered consistent with other studies (see above) showing lack of an effect of extracellular Ca^2+^ on CaSR expression within this concentration range. IL-1β acting via the IL-1 receptor decreased both PTH secretion and increased CaSR mRNA expression (Toribio et al., 2003). In rats *in vivo* serum PTH and 1,25(OH)_2_D decreased significantly 12 h after intraperitoneal (ip) injection of IL-1β, followed by significantly decreased levels of serum Ca^2+^ at 15 h (Canaff and Hendy, 2005). In the same model, decreases in serum PTH and 1,25(OH)_2_D_3_ and calcium occurred after ip injection of IL-6 (Canaff et al., 2008b).

The CaSR in part mediates the antiproliferative and prodifferentiation actions of Ca^2+^ in colonocytes and can be considered as a tumor suppressor in the colon. Fetahu et al. (2014b) showed that in two differentiated colonocyte cell lines CaSR mRNA and protein levels increased in response to the cytokines, TNFα, IL-1β, and IL-6. The robust increase of CaSR expression could represent a defense against inflammation similar to what had been shown in murine macrophages, in which lipopolysaccharide-induced TNFα release upregulated CaSR leading to negative feedback inhibition of synthesis of the cytokine (Kelly et al., 2011).

Proinflammatory cytokines, like IL-1β and TNF-α, work through NF-κB (nuclear factor kappa-light-chain-enhancer of activated B cells). NF-κB exists in the cytoplasm of cells in an inactive form bound to an inhibitor, IκB. Upon receipt of a signal generated by activation of cytokine receptors, IκB is phosphorylated, and NF-κB is released from IκB and translocates to the nucleus to upregulate specific gene transcription via specific κB response elements. NF-κB is responsible for the expression of many immune and inflammatory response (and other) proteins (Ghosh et al., 1998; Baldwin, 2001).

In the rat *in vivo* IL-1β upregulates parathyroid, thyroid, and kidney CASR mRNA and protein levels and stimulates endogenous *CASR* gene transcription in human thyroid and kidney cell lines (Canaff and Hendy, 2005). In addition, we showed that IL-1β and TNF-α upregulate *CASR* gene transcription via NF-κB with functional κB response elements being present in both promoters of the *CASR* gene (Canaff and Hendy, 2005).

At the cell surface, IL-6 binds the IL-6 receptor (IL-6R) consisting of an IL-6 binding α chain (gp80) and the gp130 signal transducer that is shared among the IL-6-related cytokine subfamily members. IL-6 binding to its receptor activates Janus kinase (JAK) family members that phosphorylate and activate signal transducers and activators of transcription (STAT) family members. The STATS dimerize, translocate to the nucleus and bind specific gene STAT-response elements activating transcription (Horvath, 2000; Levy and Darnell, 2002). STAT-1 and STAT-3 are responsible for IL-6 signaling. The JAKS may also couple to the MAPK pathway to modify the activity of transcription factors including STATs and others (Heinrich et al., 2003).

Intraperitoneal injection of IL-6 to rats *in vivo* caused decreases in serum PTH, 1,25(OH)_2_D, and Ca^2+^ that were maintained over 24 h (Canaff et al., 2008b). Parathyroid, thyroid and kidney CaSR mRNA and protein levels were upregulated. IL-6 increased *CASR* gene transcription in human thyroid C-cell and kidney proximal tubule cells *in vitro*. *CASR* gene P1 and P2 promoter-driven transcripts were upregulated. Activation of the MAPK pathway contributed modestly to basal activity of both P1 and P2 promoters, but it was not involved in a major way in the IL-6 induction of either of them (Canaff et al., 2008b). For promoter P1 a STAT1/3 element downstream of the transcriptional start site accounted for the IL-6 induction. For promoter P2 that has no STAT elements, IL-6 rapidly promotes a complex containing both Sp1/3 and STAT1/3 on GC-rich elements that are clustered at the transcription start site.

The IL-6 administration to rats resulted in a more rapid decrease in serum PTH, 1,25(OH)_2_D and Ca^2+^ levels than that occurring after IL-1β administration (Canaff and Hendy, 2005; Canaff et al., 2008b). The quicker response to IL-6 may relate to this cytokine's ability to induce rapid changes in mRNA translation and protein synthesis via the eukaryotic initiation factor-4F, as has been shown in sensory neurons, for example (Melemedjian et al., 2010). While IL-6 clearly regulates *CASR* gene transcription, uncovering any contribution of the cytokine at the post-transcriptional level with respect to the resetting of the calciostat will require further study.

### Other considerations

At sites of injury or infection the inflammatory response increases circulating proinflammatory cytokines leading to increased bone resorption. This occurs by enhanced osteocytic and osteoblastic production of receptor of activated NF-κB ligand (RANKL) resulting in increased osteoclastic bone resorption that releases Ca^2+^ from the bone (see Klein et al., 2016). One purpose of the increased extracellular Ca^2+^ concentration likely relates to its action as a chemokine on the one hand to recruit macrophages to sites of cell death and on the other to play a role in amplifying the inflammatory response via stimulating the assembly of a cytoplasmic multiprotein complex inflammasome that mediates proinflammatory cytokine maturation by activation of caspase-1 (see Hendy and Canaff, 2016 for further details).

Therefore, the CaSR in parathyroid gland and kidney executes its role in Ca^2+^ homeostasis but it is also expressed in monocytes and macrophages. This allows the CaSR to play critical roles in promoting and mediating the inflammatory response to tissue injury as well as minimizing or limiting these effects via its role in systemic Ca^2+^ homeostasis. Whether there is a place for the use of CaSR allosteric modulators in relevant clinical areas, for example, in reducing the degree of hypocalcemia in critically ill patients, remains to be explored. While extracellular Ca^2+^ is an activator of the NOD-like receptor family, pyrin domain-containing protein-3 (NLRP3) inflammasome, influenza virus infection also activates this particular inflammasome with resultant production of IL-1β (Allen et al., 2009; Owen and Gale, 2009; Thomas et al., 2009). This may be relevant to the observation that ADH1 patients with gain-of-function *CASR* mutations often become symptomatic (e.g., with seizures) during periods of intercurrent illness (Hendy et al., 2009). Enhanced expression of the activated CaSR by the increased circulating IL-1β cytokine would result in extracellular Ca^2+^ levels to drop further to symptomatic levels.

An additional pathological role for Ca^2+^, CaSR, proinflammatory cytokines, and obesity has been suggested (Villarroel et al., 2014). It is proposed that Ca^2+^ activation of the CaSR in white adipose tissue preadipocytes increases proinflammatory cytokine production, proliferation and differentiation but decreased liped accumulation and adipose tissue dysfunction (Cifuentes et al., 2012). The obesity associated cytokines like TNF-α and IL-1β increase adipocyte CaSR expression (Cifuentes et al., 2010) by releasing NFκB to translocate to the nucleus and activate the *CASR* gene at κB response elements in its promoters (Canaff and Hendy, 2005). This represents a positive feed forward loop.

### Glial cells missing-2

In Drosophila the transcription factor glial cells missing (*GCM*) acts as a developmental binary switch between glia and neurones. In mammals, there are two orthologs *GCM1*/*GCMA* and *GCM2*/*GCMB* critical for parathyroid and placental development, respectively (Kim et al., 1998; Kammerer et al., 1999; Kanemura et al., 1999). The five exons of the *GCM2* gene (OMIM# 603716) on chromosome 6p24.2 encode a protein of 506 amino acids. GCM2 is expressed in the PTH-secreting cells of the parathyroid glands and is critical for their development in terrestrial vertebrates, and continues to be expressed in the adult (Günther et al., 2000; Maret et al., 2004; Okabe and Graham, 2004; Liu et al., 2007). From NH_2_ to COOH termini of the GCM2 protein there is a DNA-binding domain, transactivation domain 1, an inhibitory domain, and transactivation domain 2.

Homozygous or heterozygous inactivating mutations occur in familial isolated hypoparathyroidism (FIH) inherited in an autosomal recessive or dominant manner, respectively (Hendy and Cole, 2015). Autosomal recessive mutations include missense, stop, frameshift, and gene deletion, (e.g., Ding et al., 2001; Baumber et al., 2005; Thomée et al., 2005; Bowl et al., 2010; Tomar et al., 2010; Doyle et al., 2012). Autosomal dominant mutations include missense and single-nucleotide deletion (e.g., Mannstadt et al., 2008; Canaff et al., 2009b; Mirczuk et al., 2010; Yi et al., 2012). *In vitro* functional studies of some of these mutants have demonstrated loss of GCM response element binding and/transcriptional activity in the case of recessive mutations, as well as the ability of dominant mutants to inhibit activity of wild-type GCM2 when the two are transfected together into cells (Mannstadt et al., 2008; Canaff et al., 2009b; Mirczuk et al., 2010).

Genetic defects affecting GCM2 are rare in FIH: a study of 20 unrelated FIH cases (10 familial and 10 sporadic) found several polymorphic variants, but did not find *GCM2* mutations that segregated with the disease and/or led to loss of function (Maret et al., 2008).

The CaSR is an early parathyroid differentiation marker (Liu et al., 2007). Absence or reduction of parathyroid GCM2, as in mice null for *Gcm2* (Günther et al., 2000) or in cultured human parathyroid cells treated with GCM2 siRNA (Mizobuchi et al., 2009), correlates with lack or decreased expression of the CaSR. GCM2 transactivates the *CASR* gene via GCM response elements in promoters P1 and P2 (Canaff et al., 2008a, 2009b). Thus, GCM2 and CaSR are mechanistically linked with respect to the development of the evolutionarily related parathyroid glands (in terrestrial vertebrates) and gills (in fish) (Okabe and Graham, 2004).

v-maf musculo-aponeurotic fibrosarcoma oncogene homolog B (MafB), a transcriptional activator, is present in developing and mature parathyroid glands (Kamitani-Kawamoto et al., 2011). MafB acts downstream of GCM2 and ensures that the developing parathyroid glands properly localize between the thyroid and the thymus (Kamitani-Kawamoto et al., 2011). GCM2 associates with MafB to synergistically activate *PTH* gene expression (Kawahara et al., 2010; Kamitani-Kawamoto et al., 2011).

Haploinsufficiency of the dual zinc-finger transcription factor, GATA3, results in the congenital hypoparathyroidism-deafness-renal dysplasia (HDR) syndrome (Ali et al., 2007). *Gata3* knockout mouse embryos lack *Gcm2* expression and have gross defects in the third and fourth pharyngeal pouches including absent parathyroid-thymus primordia (Grigorieva et al., 2010). GATA3 transactivates the *GCM2* gene by binding specifically to a double-GATA-motif within the *GCM2* promoter. In addition, GATA3 cooperates with GCM2 and MafB to activate *PTH* gene expression by interacting with the ubiquitous specificity protein-1 (SP1) transcription factor (Han et al., 2015). Thus, GATA3, GCM2 and MafB are part of a critical transcriptional cascade in the parathyroid morphogenesis and CaSR and PTH expression pathway (Grigorieva and Thakker, 2011; Han et al., 2015).

The persistance of GCM2 expression in the adult parathyroid raises the question of whether its overactivity or reduced expression could play a role in the development of parathyroid hyperfunction or tumorigenesis. In support of the first notion, increased expression of GCM2 has been noted in some adenomas of primary parathyroid patients (Kebebew et al., 2004). Furthermore, the allele frequency of a common Y282D polymorphism is significantly higher in Italian cohorts of primary hyperparathyroid patients than in normal individuals and the 282D variant exhibits higher activity on a GCM element promoter than Y282 (D'Agruma et al., 2014). In support of the second supposition, reduced levels of GCM2 mRNA have been reported in adenomatous tissue from some patients with primary hyperparathyroidism (Correa et al., 2002). This may contribute to the reduced CaSR expression commonly found in these tumors that might itself contribute to dysregulated control of parathyroid cell proliferation.

### *CASR* expression and kidney stones

Evidence has been provided that CaSR polymorphisms such as R990G can increase the susceptibility to hypercalciuria and urolithiasis (Liu et al., 2015). But single nucleotide polymorphisms (SNPs) within the regulatory regions of the *CASR* gene have also been associated with kidney stone risk, for example, in primary hyperparathyroidism (Vezzoli et al., 2011). A comparison of idiopathic calcium stone formers and healthy controls revealed one SNP (rs6776158 [A>G]) located in the *CASR* P1 promoter that was associated with nephrolithiasis (Vezzoli et al., 2013). Sr^2+^ handling has been proposed to be a mirror of Ca^2+^ metabolism and reduced Sr^2+^ excretion after an oral load was observed in GG homozygous stone formers. Activity of the G allele *CASR* P1 promoter-luciferase reporter was lower (than the A allele promoter) in cell transfection studies and CaSR mRNA levels were lower in kidney medulla samples in GG homozygous individuals than A allele carriers (Vezzoli et al., 2013). Therefore, the minor allele at rs6776158 may predispose to calcium stones by decreasing transcriptional activity of the *CASR* P1 promoter and CaSR expression in kidney tubules. Claudin-14 is a member of a superfamily of proteins that regulate paracellular transport of ions and small solutes at epithelial tight junctions. A mechanistic link between *CASR* gene activity was suggested by the finding that claudin-14 mRNA levels were lower in the *CASR* rs6776158 GG homozygous subjects Therefore, in these cases the predisposition to calcium nephrolithiasis is by a mechanism independent of hypercalciuria (Vezzoli et al., 2013). This hypothesis received further support by a comparison of another *CASR* gene regulatory SNP and R990G with respect to stone risk in primary hyperparathyroidism (Vezzoli et al., 2015).

In a genome-wide association study (GWAS) of Icelandic kidney stone cases a suggestive association of (rs7627468 [A]) in a *CASR* intron1 regulatory region was found in a large population set (Oddsson et al., 2015). A strong association was found for a polymorphism (rs199565725 [delAC]) in intron 1 of the *CLDN14* gene encoding claudin-14 that might mediate a decrease in *CLDN14* gene function. These data reinforced that of the same group in an earlier study (Thorleifsson et al., 2009). The authors observed uncorrelated genome-wide significant association of variants at the *CASR* locus that also influence biochemical traits, serum total and ionized calcium, for example, but do not associate with the kidney stone disease. This implies that the risk is not mediated solely through the serum level of calcium or other biochemical trait (Oddsson et al., 2015), and provides further indirect support for the *CASR* directed Claudin-14 mechanism (Gong et al., 2012; Gong and Hou, 2014; Toka et al., 2015; Riccardi and Valenti, 2016).

### *CASR* and epigenetic modification

Altered CaSR expression occurs in benign and malignant tumors suggesting it as either a tumor suppressor or oncogene. The epigenetic inactivation of the *CASR* may underlie the reduced CaSR expression in some cancers. The *CASR* P2 promoter that is GC-rich, is methylated to a greater extent in colorectal tumors relative to adjacent mucosa and this correlates with the reduced CaSR levels in the tumors (Hizaki et al., 2011; Fetahu et al., 2014a). Use of histone deacetylase inhibitors in colon cancer cell lines most of which poorly express CaSR has implicated the involvement of H3K9 deacetylation in the silencing of CASR in colorectal cancer (Hizaki et al., 2011; Fetahu et al., 2014a). Similar findings with respect to methylation of the *CASR* P2 promoter were reported for unfavorable neuroblastomas in which CaSR is barely detectable (Casalà et al., 2013). In addition, monosomy of chromosome 3 where *CASR* resides was found in the vast majority of primary neuroblastic tumors of all types (Casalà et al., 2013). Hence the *CASR* is silenced by both genetic and epigenetic means in these tumors. However, no evidence has been found of loss of *CASR* alleles (Farnebo et al., 1997) or *CASR* gene methylation being responsible for the reduced CaSR levels in primary and secondary hyperparathyroidism (Hofman-Bang et al., 2012; Sulaiman et al., 2013; Varshney et al., 2013). Moreover, the molecular mechanisms underlying the enhanced expression of CaSR in breast and prostate tumors that implicates an oncogenic role for CaSR in these tissues have yet to be deciphered (Tennakoon et al., 2016).

### CaSR and MicroRNAs

MicroRNAs (miRNAs) are short non-coding RNAs that function in RNA silencing and post-transcriptional regulation of gene expression. A further cause of silencing of the CaSR in colorectal tumors has been proposed to be increased expression of miR-135b and miR-146b that are considered to be oncogenic (Fetahu et al., 2016). In colon cancer cell lines other miRNAs, miR-21, miR-145, and miR-135a, are inversely correlated with CaSR expression (Singh and Chakrabarty, 2013; Singh et al., 2013). Altered expression of miRNAs may well have an important role in development of parathyroid tumors and miRNAs have been implicated in parathyroid function. Dicer-dependent maturation of parathyroid miRNAs is responsible for the dysregulated control of PTH secretion in a uremic mouse model of secondary hyperparathyroidism (Shilo et al., 2015). However, specific miRNAs that directly affect parathyroid CaSR expression have yet to be identified.

## Perspectives and conclusions

The CaSR is expressed in the central nervous system and the roles it may play in the brain are being explored (Ruat and Traiffort, 2013). It will be important in future to understand the precise mechanisms underlying the postnatal upregulation of the brain CaSR during development and whether altered regulation of the CaSR plays a role in some cases of epilepsy. Within neurons the CaSR may play a role in susceptibility to Alzheimer's Disease (AD) and its progression (Chiarini et al., 2016). *CASR* expression may be altered in AD as it is regulated by the same transcription factors (e.g., SP1/3, AP1, STAT1/3, NF-κB, TFIID) already known to modulate other AD-regulated genes (Chiarini et al., 2016). However, this has yet to be directly evaluated.

The blood-brain barrier defends extracellular Ca^2+^ in the brain from changes in serum Ca^2+^ and at rest brain extracellular Ca^2+^ is maintained at 1.1 mM. During neuronal activity extracellular Ca^2+^ can fall sharply as Ca^2+^ moves to intracellular compartments. Following action potentials, Ca^2+^ can fall as low as 0.3 mM in the synaptic cleft (Jones and Smith, 2016). Any potential changes on *CASR* gene expression and their consequences have yet to be examined.

Further insights to those described in the present review, can be anticipated with respect to identification of additional transcriptional factors and their cis-elements in the *CASR* gene promoters, epigenetic changes involving direct methylation of the *CASR* DNA as well as histone modifications and chromatin remodeling. The direct effect of miRNAs on degradation of CaSR mRNA or inhibition of its translation will become clearer.

## Author contributions

All authors listed, have made substantial, direct and intellectual contribution to the work, and approved it for publication.

### Conflict of interest statement

The authors declare that the research was conducted in the absence of any commercial or financial relationships that could be construed as a potential conflict of interest.

## References

[B1] Aida K. Koishi S. Tawata M. Onaya T. (1995). Molecular cloning of a putative Ca^2+^-sensing receptor cDNA from human kidney. Biochem. Biophys. Res. Commun. 214, 524–529. 10.1006/bbrc.1995.2318 7677761

[B2] Alam M. U. Kirton J. P. Wilkinson F. L. Towers E. Sinha S. Rouhi M.. (2009). Calcification is associated with loss of functional calcium-sensing receptor in vascular smooth muscle cells. Cardiovas. Res. 81, 260–268. 10.1093/cvr/cvn279 18852253

[B3] Ali A. Christie P. T. Grigorieva I. V. Harding B. Van Esch H. Ahmed S. F.. (2007). Functional characterization of GATA3 mutations causing the hypoparathyroidism-deafness-renal (HDR) dysplasia syndrome: insight into mechanisms of DNA binding by the GATA3 transcription factor. Hum. Mol. Genet. 16, 265–275. 10.1093/hmg/ddl454 17210674

[B4] Allen I. C. Scull M. A. Moore C. B. Holl E. K. McElvania-TeKippe E. Taxman D. J.. (2009). The NLRP3 inflammasome mediates *in vivo* innate immunity to influenza A virus through recognition of viral RNA. Immunity 30, 556–565. 10.1016/j.immuni.2009.02.005 19362020 PMC2803103

[B5] Angeletti R. H. D'Amico T. Ashok S. Russell J. (1998). The chemokine interleukin-8 regulates parathyroid secretion. J. Bone Miner. Res. 13, 1232–1237. 10.1359/jbmr.1998.13.8.1232 9718190

[B6] Bai S. Favus M. J. (2006). Vitamin D and calcium receptors: links to hypercalciuria. Curr. Opin. Nephrol. Hypertens. 15, 381–385. 10.1097/01.mnh.0000232878.50716.26 16775452

[B7] Baldwin A. S. (2001). Series introduction: the transcription factor NF-κB and human disease. J. Clin. Invest. 107, 3–6. 10.1172/JCI11891 11134170 PMC198555

[B8] Baumber L. Tufarelli C. Patel S. King P. Johnson C. A. Maher E. R.. (2005). Identification of a novel mutation disrupting the DNA binding activity of GCM2 in autosomal recessive familial isolated hypoparathyroidism. J. Med. Genet. 42, 443–448. 10.1136/jmg.2004.026898 15863676 PMC1736051

[B9] Becker K. L. Snider R. Nylen E. S. (2010). Procalcitonin in sepsis and systemic inflammation: a harmful biomarker and a therapeutic target. Br. J. Pharmacol. 159, 253–264. 10.1111/j.1476-5381.2009.00433.x 20002097 PMC2825349

[B10] Becker K. Nylen E. White J. Muller B. Snider R. Jr. (2004). Procalcitonin and the calcitonin gene family of peptides in inflammation, infection, and sepsis: a journey from calcitonin back to its precursors. J. Clin. Endocrinol. Metab. 89, 1512–1525. 10.1210/jc.2002-021444 15070906

[B11] Bowl M. R. Mirczuk S. M. Grigorieva I. V. Piret S. E. Cranston T. Southam L.. (2010). Identification and characterization of novel parathyroid-specific transcription factor Glial Cells Missing Homolog B (GCMB) mutations in eight families with autosomal recessive hypoparathyroidism. Hum. Mol. Genet. 19, 2028–2038. 10.1093/hmg/ddq084 20190276

[B12] Bradbury R. A. Sunn K. L. Crossley M. Bai M. Brown E. M. Delbridge L.. (1998). Expression of the parathyroid Ca(2+)-sensing receptor in cytotrophoblasts from human term placenta. J. Endocrinol. 156, 425–430. 10.1677/joe.0.1560425 9582498

[B13] Breitwieser G. E. (2013). The calcium sensing receptor life cycle: trafficking, cell surface expression, and degradation. Best Pract. Res. Clin. Endocrinol. Metab. 27, 303–313. 10.1016/j.beem.2013.03.003 23856261

[B14] Brennan S. C. Thiem U. Roth S. Aggarwal A. Fetahu I. Tennakoon S.. (2013). Calcium sensing receptor signalling in physiology and cancer. Biochim. Biophys. Acta 1833, 1732–1744. 10.1016/j.bbamcr.2012.12.011 23267858

[B15] Brown A. Zhong M. Finch J. Ritter C. McCracken R. Morrissey J.. (1996). Rat calcium-sensing receptor is regulated by vitamin D but not by calcium. Am. J. Physiol. 270, F454–F460. 8780248 10.1152/ajprenal.1996.270.3.F454

[B16] Brown E. M. (2013). Role of the calcium-sensing receptor in extracellular calcium homeostasis. Best Pract. Res. Clin. Endocrinol. Metab. 27, 333–343. 10.1016/j.beem.2013.02.006 23856263

[B17] Brown E. M. Gamba G. Riccardi D. Lombardi M. Butters R. Kifor O. (1993). Cloning and characterization of an extracellular Ca^2+^-sensing receptor from bovine parathyroid. Nature 366, 575–580. 8255296 10.1038/366575a0

[B18] Caldwell F. T. Jr. Graves D. B. Wallace B. H. (1997). Pathogenesis of fever in a rat burn model: the role of cytokines and lipopolysaccharide. J. Burn Care Res. 18, 525–530. 10.1097/00004630-199711000-00010 9404987

[B19] Canaff L. (2004). Extracellular Calcium-Sensing Receptor: Studies of Gene Expression and Regulation. Doctoral dissertation, McGill University.

[B20] Canaff L. Hendy G. N. (2002). Human calcium-sensing receptor gene. Vitamin D response elements in promoters P1 and P2 confer transcriptional responsiveness to 1, 25-dihydroxyvitamin D. J. Biol. Chem. 277, 30337–30350. 10.1074/jbc.M201804200 12036954

[B21] Canaff L. Hendy G. N. (2005). Calcium-sensing receptor gene transcription Is up-regulated by the proinflammatory cytokine, interleukin-1β: role of the NF-κB pathway and κB elements. J. Biol. Chem. 280, 14177–14188. 10.1074/jbc.M408587200 15684428

[B22] Canaff L. Petit J.-L. Kisiel M. Watson P. H. Gascon-Barré M. Hendy G. N. (2001). Extracellular calcium-sensing receptor is expressed in rat hepatocytes coupling to intracellular calcium mobilization and stimulation of bile flow. J. Biol. Chem. 276, 4070–4079. 10.1074/jbc.M009317200 11071898

[B23] Canaff L. Togola D. Goltzman D. Hendy G. N. (2009a). Regulation of calcium-sensing receptor gene expression by 1,25-dihydroxyvitamin D *in vivo*: studies in mice lacking the 25-hydroxyvitamin D-1alpha-hydroxylase or vitamin D receptor genes, in ASBMR 31st Annual Meeting (Denver, CO).

[B24] Canaff L. Zhou X. Cole D. E. C. Hendy G. N. (2008a). Glial cells missing-2 (GCM-2), the regulator of parathyroid cell fate, transactivates the calcium-sensing receptor gene (*CASR*): identification of GCM-response elements in *CASR* promoters P1 and P2 [Abstract M187. P.S429]. J. Bone Miner. Res. 23S1.

[B25] Canaff L. Zhou X. Hendy G. N. (2008b). The proinflammatory cytokine, interleukin-6, up-regulates calcium-sensing receptor gene transcription via Stat1/3 and Sp1/3. J. Biol. Chem. 283, 13586–13600. 10.1074/jbc.M708087200 18348986

[B26] Canaff L. Zhou X. Mosesova I. Cole D. E. Hendy G. N. (2009b). Glial Cells Missing-2 (GCM2) transactivates the calcium-sensing receptor gene: effect of a dominant-negative GCM2 mutant associated with autosomal dominant hypoparathyroidism. Hum. Mutat. 30, 85–92. 10.1002/humu.20827 18712808

[B27] Carlstedt E. Ridefelt P. Lind L. Rastad J. (1999). Interleukin-6 induced suppression of bovine parathyroid hormone secretion. Biosci. Rep. 19, 35–42. 10.1023/A:1020146023812 10379905

[B28] Casalà C. Gil-Guiñón E. Ordóñez J. L. Miguel-Queralt S. Rodríguez E. Galván P.. (2013). The calcium-sensing receptor is silenced by genetic and epigenetic mechanisms in unfavorable neuroblastomas and its reactivation induces ERK1/2-dependent apoptosis. Carcinogenesis 34, 268–276. 10.1093/carcin/bgs338 23108190

[B29] Cetani F. Picone A. Cerrai P. Vignali E. Borsari S. Pardi E. (2000). Parathyroid expression of calcium-sensing receptor protein and *in vivo* parathyroid hormone-Ca^2+^ set-point in patients with primary hyperparathyroidism. J. Clin. Endocrinol. Metab. 85, 4789–4794. 10.1210/jc.85.12.4789 11134144

[B30] Chakrabarty S. Wang H. Canaff L. Hendy G. N. Appelman H. Varani J. (2005). Calcium sensing receptor in human colon carcinoma: interaction with Ca^2+^ and 1, 25-dihydroxyvitamin D3. Cancer Res. 65, 493–498. 15695391

[B31] Chang W. Tu C. Chen T.-H. Bikle D. Shoback D. (2008). The extracellular calcium-sensing receptor (CaSR) is a critical modulator of skeletal development. Sci. Signal. 1:ra1. 10.1126/scisignal.1159945 18765830 PMC3538864

[B32] Chiarini A. Armato U. Liu D. Dal Prà I. (2016). Calcium-sensing receptors of human neural cells play crucial roles in Alzheimer's Disease. Front. Physiol. 7:134. 10.3389/fphys.2016.00134 27199760 PMC4844916

[B33] Chikatsu N. Fukumoto S. Takeuchi Y. Suzawa M. Obara T. Matsumoto T.. (2000). Cloning and characterization of two promoters for the human calcium-sensing receptor (CaSR) and changes of CaSR expression in parathyroid adenomas. J. Biol. Chem. 275, 7553–7557. 10.1074/jbc.275.11.7553 10713061

[B34] Cifuentes M. Fuentes C. Mattar P. Tobar N. Hugo E. Ben-Jonathan N.. (2010). Obesity-associated proinflammatory cytokines increase calcium sensing receptor (CaSR) protein expression in primary human adipocytes and LS14 human adipose cell line. Arch. Biochem. Biophys. 500, 151–156. 10.1016/j.abb.2010.05.033 20595056

[B35] Cifuentes M. Fuentes C. Tobar N. Acevedo I. Villalobos E. Hugo E.. (2012). Calcium sensing receptor activation elevates proinflammatory factor expression in human adipose cells and adipose tissue. Mol. Cell. Endocrinol. 361, 24–30. 10.1016/j.mce.2012.03.006 22449852 PMC3761973

[B36] Corbetta S. Mantovani G. Lania A. Borgato S. Vicentini L. Faglia G.. (2000). Calcium-sensing receptor expression and signalling in human parathyroid adenomas and primary hyperplasia. Clin. Endocrinol. (Oxf). 52, 339–348. 10.1046/j.1365-2265.2000.00933.x 10718832

[B37] Correa P. Akerström G. Westin G. (2002). Underexpression of Gcm2, a master regulatory gene of parathyroid gland development, in adenomas of primary hyperparathyroidism. Clin. Endocrinol. (Oxf). 57, 501–505. 10.1046/j.1365-2265.2002.01627.x 12354132

[B38] D'Agruma L. Coco M. Guarnieri V. Battista C. Canaff L. Salcuni A. S.. (2014). Increased prevalence of the GCM2 polymorphism, Y282D, in primary hyperparathyroidism: analysis of three Italian cohorts. J. Clin. Endocrinol. Metab. 99, E2794–E2798. 10.1210/jc.2014-2857 25279501

[B39] D'souza-Li L. Canaff L. Janicic N. Cole D. E. Hendy G. N. (2001). An acceptor splice site mutation in the calcium-sensing receptor (CASR) gene in familial hypocalciuric hypercalcemia and neonatal severe hyperparathyroidism. Hum. Mutat. 18, 411–421. 10.1002/humu.1212 11668634

[B40] Ding C. Buckingham B. Levine M. A. (2001). Familial isolated hypoparathyroidism caused by a mutation in the gene for the transcription factor GCMB. J. Clin. Invest. 108, 1215–1220. 10.1172/JCI13180 11602629 PMC209530

[B41] Doyle D. Kirwin S. M. Sol-Church K. Levine M. A. (2012). A novel mutation in the GCM2 gene causing severe congenital isolated hypoparathyroidism. J. Pediatr. Endocrinol. Metab. 25, 741–746. 10.1515/jpem-2012-0080 23155703 PMC3694175

[B42] Ekenstam E. A. Benson L. Hallgren R. Wide L. Ljunghall S. (1990). Impaired secretion of parathyroid hormone in patients with rheumatoid arthritis: relationship to inflammatory activity. Clin. Endocrinol. (Oxf). 32, 323–328. 10.1111/j.1365-2265.1990.tb00873.x 2111747

[B43] Farnebo F. Enberg U. Grimelius L. Bäckdahl M. Schalling M. Larsson C. (1997). Tumor-specific decreased expression of calcium sensing receptor messenger ribonucleic acid in sporadic primary hyperparathyroidism 1. J. Clin. Endocrinol. Metab. 82, 3481–3486. 10.1210/jc.82.10.3481 9329389

[B44] Fetahu I. S. Höbaus J. Aggarwal A. Hummel D. M. Tennakoon S. Mesteri I.. (2014a). Calcium-sensing receptor silencing in colorectal cancer is associated with promoter hypermethylation and loss of acetylation on histone 3. Int. J. Cancer 135, 2014–2023. 10.1002/ijc.28856 24691920 PMC4282356

[B45] Fetahu I. S. Hummel D. M. Manhardt T. Aggarwal A. Baumgartner-Parzer S. Kállay E. (2014b). Regulation of the calcium-sensing receptor expression by 1,25-dihydroxyvitamin D3, interleukin-6, and tumor necrosis factor alpha in colon cancer cells. J. Steroid Biochem. Mol. Biol. 144(Pt A), 228–2231. 10.1016/j.jsbmb.2013.10.015 24176760 PMC4220008

[B46] Fetahu I. S. Tennakoon S. Lines K. E. Gröschel C. Aggarwal A. Mesteri I.. (2016). miR-135b- and miR-146b-dependent silencing of calcium-sensing receptor expression in colorectal tumors. Int. J. Cancer 138, 137–145. 10.1002/ijc.29681 26178670

[B47] Freichel M. Zink-Lorenz A. Holloschi A. Hafner M. Flockerzi V. Raue F. (1996). Expression of a calcium-sensing receptor in a human medullary thyroid carcinoma cell line and its contribution to calcitonin secretion. Endocrinology 137, 3842–3848. 10.1210/endo.137.9.8756555 8756555

[B48] Garrett J. E. Capuano I. V. Hammerland L. G. Hung B. C. Brown E. M. Hebert S. C.. (1995). Molecular cloning and functional expression of human parathyroid calcium receptor cDNAs. J. Biol. Chem. 270, 12919–12925. 10.1074/jbc.270.21.12919 7759551

[B49] Ghosh S. May M. J. Kopp E. B. (1998). NF-kappa B and Rel proteins: evolutionarily conserved mediators of immune responses. Annu. Rev. Immunol. 16, 225–260. 10.1146/annurev.immunol.16.1.225 9597130

[B50] Gogusev J. Duchambon P. Hory B. Giovannini M. Goureau Y. Sarfati E.. (1997). Depressed expression of calcium receptor in parathyroid gland tissue of patients with hyperparathyroidism. Kidney Int. 51, 328–336. 10.1038/ki.1997.41 8995751

[B51] Goltzman D. Hendy G. N. (2015). The calcium-sensing receptor in bone – mechanistic and therapeutic insights. Nat. Rev. Endocrinol. 11, 298–307. 10.1038/nrendo.2015.30 25752283

[B52] Gong Y. Hou J. (2014). Claudin-14 underlies Ca^++^-sensing receptor-mediated Ca^++^ metabolism via NFAT-microRNA-based mechanisms. J. Am. Soc. Nephrol. 25, 745–760. 10.1681/ASN.2013050553 24335970 PMC3968499

[B53] Gong Y. Renigunta V. Himmerkus N. Zhang J. Renigunta A. Bleich M.. (2012). Claudin-14 regulates renal Ca^++^ transport in response to CaSR signalling via a novel microRNA pathway. EMBO J. 31, 1999–2012. 10.1038/emboj.2012.49 22373575 PMC3343334

[B54] Grigorieva I. V. Mirczuk S. Gaynor K. U. Nesbit M. A. Grigorieva E. F. Wei Q.. (2010). Gata3-deficient mice develop parathyroid abnormalities due to dysregulation of the parathyroid-specific transcription factor Gcm2. J. Clin. Invest. 120, 2144–2155. 10.1172/JCI42021 20484821 PMC2877956

[B55] Grigorieva I. V. Thakker R. V. (2011). Transcription factors in parathyroid development: lessons from hypoparathyroid disorders. Ann. N. Y. Acad. Sci. 1237, 24–38. 10.1111/j.1749-6632.2011.06221.x 22082362

[B56] Günther T. Chen Z. F. Kim J. Priemel M. Rueger J. M. Amling M.. (2000). Genetic ablation of parathyroid glands reveals another source of parathyroid hormone. Nature 406, 199–203. 10.1038/35018111 10910362

[B57] Guo Y. Dickerson C. Chrest F. J. Adler W. H. Munster A. M. Winchurch R. A. (1990). Increased levels of circulating interleukin 6 in burn patients. Clin. Immunol. Immunopathol. 54, 361–371. 10.1016/0090-1229(90)90050-Z 2406054

[B58] Hannan F. M. Thakker R. V. (2013). Calcium-sensing receptor (CaSR) mutations and disorders of calcium, electrolyte and water metabolism. Best Pract. Res. Clin. Endocrinol. Metab. 27, 359–371. 10.1016/j.beem.2013.04.007 23856265

[B59] Han S.-I. Tsunekage Y. Kataoka K. (2015). Gata3 cooperates with Gcm2 and MafB to activate parathyroid hormone gene expression by interacting with SP1. Mol. Cell. Endocrinol. 411, 113–120. 10.1016/j.mce.2015.04.018 25917456

[B60] Heinrich P. C. Behrmann I. Haan S. Hermanns H. M. Müller-Newen G. Schaper F. (2003). Principles of interleukin (IL)-6-type cytokine signalling and its regulation. Biochem. J. 374, 1–20. 10.1042/bj20030407 12773095 PMC1223585

[B61] Hénaut L. Boudot C. Massy Z. A. Lopez-Fernandez I. Dupont S. Mary A.. (2014). Calcimimetics increase CaSR expression and reduce mineralization in vascular smooth muscle cells: mechanisms of action. Cardiovasc. Res. 101, 256–265. 10.1093/cvr/cvt249 24217682

[B62] Hendy G. N. Canaff L. (2016). Calcium-sensing receptor, proinflammatory cytokines and calcium homeostasis. Semin. Cell. Dev. Biol. 49, 37–43. 10.1016/j.semcdb.2015.11.006 26612442

[B63] Hendy G. N. Canaff L. Cole D. E. (2013). The CASR gene: alternative splicing and transcriptional control, and calcium-sensing receptor (CaSR) protein: structure and ligand binding sites. Best Pract. Res. Clin. Endocrinol. Metab. 27, 285–301. 10.1016/j.beem.2013.02.009 23856260

[B64] Hendy G. N. Cole D. E. C. (2015). Familial isolated hypoparathyroidism, in Hypoparathyroidism, eds Brandi M. L. Brown E. M. (Springer-Verlag Italia), 167–175.

[B65] Hendy G. N. Guarnieri V. Canaff L. (2009). Calcium-sensing receptor and associated diseases. Prog. Mol. Biol. Transl. Sci. 89, 31–95. 10.1016/S1877-1173(09)89003-0 20374733

[B66] Hizaki K. Yamamoto H. Taniguchi H. Adachi Y. Nakazawa M. Tanuma T.. (2011). Epigenetic inactivation of calcium-sensing receptor in colorectal carcinogenesis. Mod. Pathol. 24, 876–884. 10.1038/modpathol.2011.10 21317879

[B67] Ho C. Conner D. A. Pollak M. R. Ladd D. J. Kifor O. Warren H. B.. (1995). A mouse model of human familial hypocalciuric hypercalcemia and neonatal severe hyperparathyroidism. Nat. Genet. 11, 389–394. 10.1038/ng1295-389 7493018

[B68] Hofman-Bang J. Gravesen E. Olgaard K. Lewin E. (2012). Epigenetic methylation of parathyroid CaR and VDR promoters in experimental secondary hyperparathyroidism. Int. J. Nephrol. 2012:123576. 10.1155/2012/123576 23094155 PMC3474253

[B69] Horvath C. M. (2000). STAT proteins and transcriptional responses to extracellular signals. Trends Biochem. Sci. 25, 496–502. 10.1016/S0968-0004(00)01624-8 11050435

[B70] Hurcombe S. D. Toribio R. E. Slovis N. M. Saville W. J. Mudge M. C. Macgillivray K.. (2009). Calcium regulating hormones and serum calcium and magnesium concentrations in septic and critically ill foals and their association with survival. J. Vet. Intern. Med. 23, 335–343. 10.1111/j.1939-1676.2009.0275.x 19210311

[B71] Imanishi Y. Kawata T. Kenko T. Wada M. Nagano N. Miki T.. (2011). Cinacalcet HCl suppresses Cyclin D1 oncogene-derived parathyroid cell proliferation in a murine model for primary hyperparathyroidism. Calcif. Tissues Int. 89, 29–35. 10.1007/s00223-011-9490-4 21541686

[B72] Janicic N. Soliman E. Pausova Z. Seldin M. F. Rivière M. Szpirer J.. (1995). Mapping of the calcium-sensing receptor gene (CASR) to human chromosome 3q13. 3-21 by fluorescence *in situ* hybridization, and localization to rat chromosome 11 and mouse chromosome 16. Mamm. Genome 6, 798–801. 8597637 10.1007/BF00539007

[B73] Jones B. L. Smith S. M. (2016). Calcium-sensing receptor: a key target for extracellular calcium signaling in neurons. Front. Physiol. 7:116. 10.3389/fphys.2016.00116 27065884 PMC4811949

[B74] Kállay E. Bonner E. Wrba F. Thakker R. V. Peterlik M. Cross H. S. (2003). Molecular and functional characterization of the extracellular calcium-sensing receptor in human colon cancer cells. Oncol. Res. Featuring Preclin. Clin. Cancer Ther. 13, 551–559. 12899245 10.3727/000000003108748072

[B75] Kamitani-Kawamoto A. Hamada M. Moriguchi T. Miyai M. Saji F. Hatamura I.. (2011). MafB interacts with Gcm2 and regulates parathyroid hormone expression and parathyroid development. J. Bone Miner. Res. 26, 2463–2472. 10.1002/jbmr.458 21713993

[B76] Kammerer M. Pirola B. Giglio S. Giangrande A. (1999). GCMB, a second human homolog of the fly glide/gcm gene. Cytogenet. Cell Genet. 84, 43–47. 10343099 10.1159/000015210

[B77] Kanemura Y. Hiraga S. Arita N. Ohnishi T. Izumoto S. Mori K.. (1999). Isolation and expression analysis of a novel human homologue of the Drosophila glial cells missing (gcm) gene. FEBS Lett. 442, 151–156. 9928992 10.1016/s0014-5793(98)01650-0

[B78] Katz M. S. Gutierrez G. E. Mundy G. R. Caulfield M. P. Hymer T. K. McKee R. L. (1992). Tumor necrosis factor and interleukin 1 inhibit parathyroid hormone-responsive adenylate cyclase in clonal osteoblast-like cell by down-regulating parathyroid hormone receptors. J. Cell. Physiol. 153, 206–213. 10.1002/jcp.1041530125 1325978

[B79] Kawahara M. Iwasaki Y. Sakaguchi K. Taguchi T. Nishiyama M. Nigawara T.. (2010). Involvement of GCMB in the transcriptional regulation of the human parathyroid hormone gene in a parathyroid-derived cell line PT-r: effects of calcium and 1,25(OH)2D3. Bone 47, 534–541. 10.1016/j.bone.2010.05.031 20558332

[B80] Kawata T. Imanishi Y. Kobayashi K. Kenko T. Wada M. Ishimura E.. (2005). Relationship between parathyroid calcium-sensing receptor expression and potency of the calcimimetic, cinacalcet, in suppressing parathyroid hormone secretion in an *in vivo* murine model of primary hyperparathyroidism. Eur. J. Endocrinol. 153, 587–594. 10.1530/eje.1.02007 16189180

[B81] Kebebew E. Peng M. Wong M. G. Ginzinger D. Duh Q.-Y. Clark O. H. (2004). GCMB gene, a master regulator of parathyroid gland development, expression, and regulation in hyperparathyroidism. Surgery 136, 1261–1266. 10.1016/j.surg.2004.06.056 15657585

[B82] Kelly J. C. Lungchukiet P. MacLeod R. J. (2011). Extracellular calcium-sensing receptor inhibition of intestinal epithelial TNF signaling requires CaSR-mediated Wnt5a/Ror2 interaction. Front. Physiol. 2:17. 10.3389/fphys.2011.00017 21603229 PMC3093814

[B83] Kifor O. Moore F. Jr. Wang P. Goldstein M. Vassilev P. Kifor I.. (1996). Reduced immunostaining for the extracellular Ca^2+^-sensing receptor in primary and uremic secondary hyperparathyroidism. J. Clin. Endocrinol. Metab. 81, 1598–1606. 8636374 10.1210/jcem.81.4.8636374

[B84] Kim J. Jones B. W. Zock C. Chen Z. Wang H. Goodman C. S.. (1998). Isolation and characterization of mammalian homologs of the Drosophila gene glial cells missing. Proc. Natl. Acad. Sci. U.S.A. 95, 12364–12369. 9770492 10.1073/pnas.95.21.12364 PMC22837

[B85] Klein G. L. Castro S. M. Garofalo R. P. (2016). The calcium-sensing receptor as a mediator of inflammation. Semin. Cell. Dev. Biol. 49, 52–56. 10.1016/j.semcdb.2015.08.006 26303192 PMC4761504

[B86] Klein G. L. Herndon D. N. Goodman W. G. Langman C. B. Phillips W. A. Dickson I. R.. (1995). Histomorphometric and biochemical characterization of bone following acute severe burns in children. Bone 17, 455–460. 10.1016/8756-3282(95)00279-1 8579956

[B87] Klein G. L. Nicolai M. Langman C. B. Cuneo B. F. Sailer D. E. Herndon D. N. (1997). Dysregulation of calcium homeostasis after severe burn injury in children: possible role of magnesium depletion. J. Pediatr. 131, 246–251. 10.1016/S0022-3476(97)70161-6 9290611

[B88] Kowal-Vern A. Walenga J. M. Hoppensteadt D. Sharp-Pucci M. Gamelli R. L. (1994). Interleukin-2 and interleukin-6 in relation to burn wound size in the acute phase of thermal injury. J. Am. Coll. Surg. 178, 357–362. 8149035

[B89] Lepage R. Légaré G. Racicot C. Brossard J.-H. Lapointe R. Dagenais M.. (1999). Hypocalcemia induced during major and minor abdominal surgery in humans. J. Clin. Endocrinol. Metab. 84, 2654–2658. 10.1210/jcem.84.8.5889 10443655

[B90] Levy D. E. Darnell J. (2002). Stats: transcriptional control and biological impact. Nat. Rev. Mol. Cell Biol. 3, 651–662. 10.1038/nrm909 12209125

[B91] Lienhardt A. Bai M. Lagarde J.-P. Rigaud M. Zhang Z. Jiang Y.. (2001). Activating mutations of the calcium-sensing receptor: management of hypocalcemia. J. Clin. Endocrinol. Metab. 86, 5313–5323. 10.1210/jcem.86.11.8016 11701698

[B92] Lind L. Carlstedt F. Rastad J. Stiernström H. Stridsberg M. Ljunggren Ö.. (2000). Hypocalcemia and parathyroid hormone secretion in critically ill patients. Crit. Care Med. 28, 93–99. 10.1097/00003246-200001000-00015 10667505

[B93] Liu K. Wang X. Ye J. Qin C. Shao P. Zhang W.. (2015). The G allele of CaSR R990G polymorphism increases susceptibility to urolithiasis and hypercalciuria: evidences from a comprehensive meta-analysis. Biomed. Res. Int. 2015:958207. 10.1155/2015/958207 25705702 PMC4331470

[B94] Liu Z. Yu S. Manley N. R. (2007). Gcm2 is required for the differentiation and survival of parathyroid precursor cells in the parathyroid/thymus primordia. Dev. Biol. 305, 333–346. 10.1016/j.ydbio.2007.02.014 17382312 PMC1931567

[B95] Li X. Zheng W. Li Y. C. (2003). Altered gene expression profile in the kidney of vitamin D receptor knockout mice. J. Cell. Biochem. 89, 709–719. 10.1002/jcb.10547 12858337

[B96] Loretz 2008###Loretz, C. A. (2008). Extracellular calcium-sensing receptors in fishes. Comp. Biochem. Physiol. Part A: Mol. Integr. Physiol. 149, 225–245. 10.1016/j.cbpa.2008.01.037 18302989

[B97] Loupy A. Ramakrishnan S. K. Wootla B. Chambrey R. de la Faille R. Bourgeois S.. (2012). PTH-independent regulation of blood calcium concentration by the calcium-sensing receptor. J. Clin. Invest. 122, 3355–3367. 10.1172/JCI57407 22886306 PMC3428075

[B98] Mannstadt M. Bertrand G. Muresan M. Weryha G. Leheup B. Pulusani S. R.. (2008). Dominant-negative GCMB mutations cause an autosomal dominant form of hypoparathyroidism. J. Clin. Endocrinol. Metab. 93, 3568–3576. 10.1210/jc.2007-2167 18583467 PMC2567849

[B99] Maret A. Bourdeau I. Ding C. Kadkol S. S. Westra W. H. Levine M. A. (2004). Expression of GCMB by intrathymic parathyroid hormone-secreting adenomas indicates their parathyroid cell origin. J. Clin. Endocrinol. Metab. 89, 8–12. 10.1210/jc.2003-030733 14715818

[B100] Maret A. Ding C. Kornfield S. L. Levine M. A. (2008). Analysis of the GCM2 gene in isolated hypoparathyroidism: a molecular and biochemical study. J. Clin. Endocrinol. Metab. 93, 1426–1432. 10.1210/jc.2007-1783 18182452

[B101] Mary A. Hénaut L. Boudot C. Six I. Brazier M. Massy Z. A.. (2015). Calcitriol prevents *in vitro* vascular smooth muscle cell mineralization by regulating calcium-sensing receptor expression. Endocrinology 156, 1965–1974. 10.1210/en.2014-1744 25763635

[B102] Melemedjian O. K. Asiedu M. N. Tillu D. V. Peebles K. A. Yan J. Ertz N.. (2010). IL-6-and NGF-induced rapid control of protein synthesis and nociceptive plasticity via convergent signaling to the eIF4F complex. J. Neurosci. 30, 15113–15123. 10.1523/JNEUROSCI.3947-10.2010 21068317 PMC3056511

[B103] Mirczuk S. M. Bowl M. R. Nesbit M. A. Cranston T. Fratter C. Allgrove J.. (2010). A missense glial cells missing homolog B (GCMB) mutation, Asn502His, causes autosomal dominant hypoparathyroidism. J. Clin. Endocrinol. Metab. 95, 3512–3516. 10.1210/jc.2009-2532 20463099

[B104] Mizobuchi M. Ritter C. S. Krits I. Slatopolsky E. Sicard G. Brown A. J. (2009). Calcium-sensing receptor expression is regulated by glial cells missing-2 in human parathyroid cells. J. Bone Miner. Res. 24 1173–1179. 10.1359/jbmr.090211 19257819 PMC2697623

[B105] Molostvov G. Hiemstra T. F. Fletcher S. Bland R. Zehnder D. (2015). Arterial Expression of the calcium-sensing receptor is maintained by physiological pulsation and protects against calcification. PLoS ONE 10:e0138833. 10.1371/journal.pone.0138833 26436544 PMC4593585

[B106] Müller B. Becker K. Kränzlin M. Schachinger H. Huber P. Nylen E.. (2000). Disordered calcium homeostasis of sepsis: association with calcitonin precursors. Eur. J. Clin. Invest. 30, 823–831. 10.1046/j.1365-2362.2000.00714.x 10998084

[B107] Murphey E. Chattopadhyay N. Bai M. Kifor O. Harper D. Traber D. L.. (2000). Up-regulation of the parathyroid calcium-sensing receptor after burn injury in sheep: a potential contributory factor to postburn hypocalcemia. Crit. Care Med. 28, 3885–3890. 10.1097/00003246-200012000-00024 11153630

[B108] Naito T. Saito Y. Yamamoto J. Nozaki Y. Tomura K. Hazama M.. (1998). Putative pheromone receptors related to the Ca^2+^-sensing receptor in Fugu. Proc. Natl. Acad. Sci. U.S.A. 95, 5178–5181. 10.1073/pnas.95.9.5178 9560249 PMC20234

[B109] Nielsen P. Rasmussen A. K. Butters R. Feldt-Rasmussen U. Bendtzen K. Diaz R.. (1997). Inhibition of PTH secretion by Interleukin-1β in bovine parathyroid glands *in vitro* Is associated with an up-regulation of the Calcium-Sensing Receptor mRNA. Biochem. Biophys. Res. Commun. 238, 880–885. 10.1006/bbrc.1997.7207 9325185

[B110] Nijsten M. W. Hack C. E. Helle M. Ten Duis H. J. Klasen H. J. Aarden L. A. (1991). Interleukin-6 and its relation to the humoral immune response and clinical parameters in burned patients. Surgery 109, 761–767. 2042096

[B111] Obermannova B. Sumnik A. Dusatkova P. Cinek O. Grant M. Lebl J.. (2016). Novel calcium-sensing receptor cytoplasmic tail deletion mutation causing autosomal dominant hypocalcemia: molecular and clinical study. Eur. J. Endocrinol. 174, K1–K11. 10.1530/EJE-15-1216 26764418

[B112] Oda Y. Tu C.-L. Chang W. Crumrine D. Kömüves L. Mauro T.. (2000). The calcium sensing receptor and its alternatively spliced form in murine epidermal differentiation. J. Biol. Chem. 275, 1183–1190. 10.1074/jbc.275.2.1183 10625662

[B113] Oda Y. Tu C.-L. Pillai S. Bikle D. D. (1998). The calcium sensing receptor and its alternatively spliced form in keratinocyte differentiation. J. Biol. Chem. 273, 23344–23352. 10.1074/jbc.273.36.23344 9722568

[B114] Oddsson A. Sulem P. Helgason H. Edvardsson V. O. Thorleifsson G. Sveinbjörnsson G.. (2015). Common and rare variants associated with kidney stones and biochemical traits. Nat. Commun. 6:7975. 10.1038/ncomms8975 26272126 PMC4557269

[B115] Ohzato H. Monden M. Yoshizaki K. Ogata A. Nishimoto N. Gotoh M.. (1993). Systemic production of interleukin-6 following acute inflammation. Biochem. Biophys. Res. Commun. 197, 1556–1562. 10.1006/bbrc.1993.2655 8280175

[B116] Okabe M. Graham A. (2004). The origin of the parathyroid gland. Proc. Natl. Acad. Sci. U.S.A. 101, 17716–17719. 10.1073/pnas.0406116101 15591343 PMC539734

[B117] Owen D. M. Gale M. Jr. (2009). Fighting the flu with inflammasome signaling. Immunity 30, 476–478. 10.1016/j.immuni.2009.03.011 19371712

[B118] Pearce S. H. Williamson C. Kifor O. Bai M. Coulthard M. G. Davies M.. (1996). A familial syndrome of hypocalcemia with hypercalciuria due to mutations in the calcium-sensing receptor. N. Eng. J. Med. 335, 1115–1122. 10.1056/NEJM199610103351505 8813042

[B119] Pollak M. R. Brown E. Chou Y. Hebert S. Marx S. Steinman B. (1993). Mutations in the human Ca^2+^ sensing receptor gene cause familial hypocalciuric hypercalcemia and neonatal severe hyperparathyroidism. Cell 75, 1297–1303. 10.1016/0092-8674(93)90617-Y 7916660

[B120] Remick D. G. Bolgos G. R. Siddiqui J. Shin J. Nemzek J. A. (2002). Six at six: interleukin-6 measured 6 h after the initiation of sepsis predicts mortality over 3 days. Shock 17, 463–467. 10.1097/00024382-200206000-00004 12069181

[B121] Riccardi D. Brennan S. C. Chang W. (2013). The extracellular calcium-sensing receptor, CaSR, in fetal development. Best Pract. Res. Clin. Endocrinol. Metab. 27, 443–453. 10.1016/j.beem.2013.02.010 23856271 PMC4462341

[B122] Riccardi D. Valenti G. (2016). Localization and function of the renal calcium-sensing receptor. Nat. Rev. Nephrol. 12, 414–425. 10.1038/nrneph.2016.59 27157444

[B123] Rodriguez L. Tu C. Cheng Z. Chen T.-H. Bikle D. Shoback D.. (2005). Expression and functional assessment of an alternatively spliced extracellular Ca^2+^-sensing receptor in growth plate chondrocytes. Endocrinology 146, 5294–5303. 10.1210/en.2005-0256 16166224

[B124] Rogers K. Dunn C. Conklin R. Hadfield S. Petty B. Brown E.. (1995). Calcium receptor messenger ribonucleic acid levels in the parathyroid glands and kidney of vitamin D-deficient rats are not regulated by plasma calcium or 1, 25-dihydroxyvitamin D3. Endocrinology 136, 499–504. 7835282 10.1210/endo.136.2.7835282

[B125] Ruat M. Traiffort E. (2013). Roles of the calcium sensing receptor in the central nervous system. Best Pract. Res. Clin. Endocrinol. Metab. 27, 429–442. 10.1016/j.beem.2013.03.001 23856270

[B126] Schlüter B. König B. Bergmann U. Müller F. E. König W. (1991). Interleukin 6-a potential mediator of lethal sepsis after major thermal trauma: evidence for increased IL-6 production by peripheral blood mononuclear cells. J. Trauma Acute Care Surg. 31, 1663–1670. 10.1097/00005373-199112000-00017 1749040

[B127] Shilo V. Ben-Dov I. Z. Nechama M. Silver J. Naveh-Many T. (2015). Parathyroid-specific deletion of dicer-dependent microRNAs abrogates the response of the parathyroid to acute and chronic hypocalcemia and uremia. FASEB J. 29, 3964–3976. 10.1096/fj.15-274191 26054367

[B128] Singh N. Chakrabarty S. (2013). Induction of CaSR expression circumvents the molecular features of malignant CaSR null colon cancer cells. Int. J. Cancer 133, 2307–2314. 10.1002/ijc.28270 23674327

[B129] Singh N. Liu G. Chakrabarty S. (2013). Isolation and characterization of calcium sensing receptor null cells: a highly malignant and drug resistant phenotype of colon cancer. Int. J. Cancer 132, 1996–2005. 10.1002/ijc.27902 23055106

[B130] Steele T. Kolamunnage-Dona R. Downey C. Toh C.-H. Welters I. (2013). Assessment and clinical course of hypocalcemia in critical illness. Crit. Care 17:R106. 10.1186/cc12756 23734769 PMC4056680

[B131] Sulaiman L. Juhlin C. C. Nilsson I. L. Fotouhi O. Larsson C. Hashemi J. (2013). Global and gene-specific promoter methylation analysis in primary hyperparathyroidism. Epigenetics 8, 646–655. 10.4161/epi.24823 23764768 PMC3857344

[B132] Tennakoon S. Aggarwal A. Kállay E. (2016). The calcium-sensing receptor and the hallmarks of cancer. Biochim. Biophys. Acta 1863(6 Pt B), 1398–1407. 10.1016/j.bbamcr.2015.11.017 26608608

[B133] Thomas P. G. Dash P. Aldridge J. R. Ellebedy A. H. Reynolds C. Funk A. J.. (2009). The intracellular sensor NLRP3 mediates key innate and healing responses to influenza A virus via the regulation of caspase-1. Immunity 30, 566–575. 10.1016/j.immuni.2009.02.006 19362023 PMC2765464

[B134] Thomée C. Schubert S. W. Parma J. Lê P. Q. Hashemolhosseini S. Wegner M.. (2005). GCMB mutation in familial isolated hypoparathyroidism with residual secretion of parathyroid hormone. J. Clin. Endocrinol. Metab. 90, 2487–2492. 10.1210/jc.2004-2450 15728199

[B135] Thorleifsson G. Holm H. Edvardsson V. Walters G. B. Styrkarsdottir U. Gudbjartsson D. F.. (2009). Sequence variants in the CLDN14 gene associate with kidney stones and bone mineral density. Nat. Genet. 41, 926–930. 10.1038/ng.404 19561606

[B136] Toka H. R. Pollak M. R. Houillier P. (2015). Calcium sensing in the renal tubule. Physiology (Bethesda) 30, 317–326. 10.1152/physiol.00042.2014 26136545

[B137] Tomar N. Bora H. Singh R. Gupta N. Kaur P. Chauhan S. S.. (2010). Presence and significance of a R110W mutation in the DNA-binding domain of GCM2 gene in patients with isolated hypoparathyroidism and their family members. Eur. J. Endocrinol. 162, 407–421. 10.1530/EJE-09-0303 19940031

[B138] Toribio R. E. Kohn C. W. Capen C. C. Rosol T. J. (2003). Parathyroid hormone (PTH) secretion, PTH mRNA and calcium-sensing receptor mRNA expression in equine parathyroid cells, and effects of interleukin (IL)-1, IL-6, and tumor necrosis factor-alpha on equine parathyroid cell function. J. Mol. Endocrinol. 31, 609–620. 10.1677/jme.0.0310609 14664720

[B139] Toribio R. E. Kohn C. W. Chew D. J. Sams R. A. Rosol T. J. (2001). Comparison of serum parathyroid hormone and ionized calcium and magnesium concentrations and fractional urinary clearance of calcium and phosphorus in healthy horses and horses with enterocolitis. Am. J. Vet. Res. 62, 938–947. 10.2460/ajvr.2001.62.938 11400854

[B140] Varshney S. Bhadada S. K. Sachdeva N. Arya A. K. Saikia U. N. Behera A.. (2013). Methylation status of the CpG islands in vitamin D and calcium-sensing receptor gene promoters does not explain the reduced gene expressions in parathyroid adenomas. J. Clin. Endocrinol. Metab. 98, E1631–E1635. 10.1210/jc.2013-1699 23913941

[B141] Vezzoli G. Scillitani A. Corbetta S. Terranegra A. Dogliotti E. Guarnieri V.. (2011). Polymorphisms at the regulatory regions of the CASR gene influence stone risk in primary hyperparathyroidism. Eur. J. Endocrinol. 164, 421–427. 10.1530/EJE-10-0915 21183554

[B142] Vezzoli G. Scillitani A. Corbetta S. Terranegra A. Dogliotti E. Guarnieri V.. (2015). Risk of nephrolithiasis in primary hyperparathyroidism is associated with *two* polymorphisms of the calcium-sensing receptor gene. J. Nephrol. 28, 67–72. 10.1007/s40620-014-0106-8 24832896

[B143] Vezzoli G. Terranegra A. Aloia A. Arcidiacono T. Milanesi L. Mosca E.. (2013). Decreased transcriptional activity of calcium-sensing receptor gene promoter 1 is associated with calcium nephrolithiasis. J. Clin. Endocrinol. Metab. 98, 3839–3847. 10.1210/jc.2013-183 23864702 PMC3763974

[B144] Villarroel P. Villalobos E. Reyes M. Cifuentes M. (2014). Calcium, obesity, and the role of the calcium-sensing receptor. Nutr. Rev. 72, 627–637. 10.1111/nure.12135 25182976

[B145] Yamada Y. Endo S. Inada K. (1996). Plasma cytokine levels in patients with severe burn injury-with reference to the relationship between infection and prognosis. Burns 22, 587–593. 10.1016/S0305-4179(96)00052-6 8982534

[B146] Yao J. J. Bai S. Karnauskas A. J. Bushinsky D. A. Favus M. J. (2005). Regulation of renal calcium receptor gene expression by 1, 25-dihydroxyvitamin D3 in genetic hypercalciuric stone-forming rats. J. Am. Soc. Nephrol. 16, 1300–1308. 10.1681/ASN.2004110991 15788476

[B147] Yi H. S. Eom Y. S. Park I. B. Lee S. Hong S. Jüppner H.. (2012). Identification and characterization of C106R, a novel mutation in the DNA-binding domain of GCMB, in a family with autosomal-dominant hypoparathyroidism. Clin. Endocrinol. (Oxf). 76, 625–633. 10.1111/j.1365-2265.2011.04256.x 22066718 PMC3701386

[B148] Yun F. H. Wong B. Y. Chase M. Shuen A. Y. Canaff L. Thongthai K.. (2007). Genetic variation at the calcium-sensing receptor (CASR) locus: implications for clinical molecular diagnostics. Clin. Biochem. 40, 551–561. 10.1016/j.clinbiochem.2006.12.011 17320849

[B149] Zaloga G. P. (1992). Hypocalcemia in critically ill patients. Crit. Care Med. 20, 251–262. 10.1097/00003246-199202000-00014 1737459

[B150] Zivin J. R. Gooley T. Zager R. A. Ryan M. J. (2001). Hypocalcemia: a pervasive metabolic abnormality in the critically ill. Am. J. Kidney Dis. 37, 689–698. 10.1016/S0272-6386(01)80116-5 11273867

